# Editorial: Rare genetic disorders associated with intellectual disability

**DOI:** 10.3389/fpsyt.2026.1792382

**Published:** 2026-02-26

**Authors:** Mustafa A. Salih

**Affiliations:** Professor and Consultant Pediatric Neurologist, Health Sector, King Abdulaziz City for Science and Technology, Riyadh, Saudi Arabia

**Keywords:** intellectual disability (ID), multiple mitochondrial dysfunctions syndrome 4, NACC1 gene, pyridoxine-dependent epilepsy, quality of life of children with trisomy 21, rare genetic disorders

Intellectual disability (ID) is associated with a substantial impairment in intellectual functioning and adaptive behavior that starts in the developmental period. The prevalence of ID is about 2–3% of the global population with regional variations influenced by socioeconomic background. Based on adaptive functioning in the DSM-5, severity has been categorized into mild, moderate, severe and profound (1). Multiple exogenous and endogenous factors, many of genetic origin, are causative of ID (2, 3).

This Research Topic was designed to highlight the current state of knowledge regarding rare genetic disorders associated with ID. The goals included highlighting etiological diagnosis, identification of treatable causes, the discovery of ID genes and variants to facilitate data sharing and subsequent variant interpretation, the provision of genetic counseling and the development of prevention programs. Of the six initially submitted manuscripts by international researchers, four were considered to be suitable for publication following a thorough peer review process.

Salih et al. reported a novel pathogenic variant in the *ALDH7A1* gene, known to be associated with pyridoxine-dependent epilepsy (PDE) (4). The condition, which results from aberrant lysine degradation and accumulation of neurotoxic metabolites, is potentially fatal if untreated. It is characterized by epileptic seizures which are resistant to standard anti-seizure medications (ASM) and responds only to pyridoxine therapy.

A consanguineous family with two affected siblings was described. Patient 1, 13-year-old girl who was born normally at term, had seizures occurring 6 hours after birth which were resistant to ASM, but stopped following administration of pyridoxine (40 mg daily) 2 days later. Her development continued to be delayed and at the age of 8 years pyridoxine was increased to 100 mg daily. She managed to participate well in a regular school. Her younger sibling (Patient 2) is now a 12-year-old boy, who started to have seizures 10 hours after birth, but immediately received 40 mg of pyridoxine with subsequent control of seizures. The dose of pyridoxine was increased to 100 mg at the age of 7 years. He is currently in 5th Grade and has dyslexia.

Using whole Exome sequencing and homozygosity mapping, a novel homozygous missense variant in *ALDH7A1* gene was detected in Patients 1and 2.

The study suggested that developmental delay and ID may persist in association with PDE despite the institution of early treatment providing seizure control. The study also highlighted the currently recommended dose for the treatment of PDE and the pathogenesis of ID despite early control of seizure with pyridoxine. It also mentioned the value of lysine reduction therapy (LRD) in addition to pyridoxine to guard against the development of ID (5). This was not used in either of the two patients due to lack of feasibility. It is noteworthy that a promising genetic therapy for PDE has recently been reported which downregulates alphaaminoadipic semialdehyde synthase (AASS), the first enzyme of the lysine catabolism, leading to alleviation of the metabolic abnormalities (6, 7).

Al-Hassnan et al. reported a novel homozygous *ISCA2* missensce variant (NM_194279.3:c.70A>G:p.Arg24Gly) in a 24-month old Saudi child with progressive loss of milestones, neuroregression, diffuse pallor of the optic disc, head lag, generalized spasticity and exaggerated deep tendon reflexes. This contribution expands the novel syndrome which they previously described and which results from a homozygous pathogenic (a founder mutation among Saudis) of *ISCA2* (8). The syndrome, which is transmitted as autosomal recessive, is known as multiple mitochondrial dysfunctions syndrome 4 (MMDS4, MIM #616370). Individuals affected with MMDS4 have normal development for the first months of life but show progressive loss of motor and social skills thereafter.

The novel *ISCA2* variant reported by Al-Hassnan et al. was detected using whole exome sequencing (WES) coupled with autozygome analysis. They also assessed mitochondrial DNA (mt DNA) copy number and mt DNA sequencing on DNA extracted from blood and cultured fibroblasts. From functional studies including splicing assessment of *ISCA2* it was concluded that the variant is pathogenic through disrupting splicing of normal *ISCA2*.

Wu et al. reported a novel *de novo* heterozygous variant in the *NACC1* gene in an 18-month-old female patient who presented with profound developmental delay and had severe ID, epilepsy, and severe malnutrition. Electroencephalography (EEG) showed bilateral slow wave activity mixed with spike waves during sleep. MRI revealed reduced volume of frontal and parietal lobes associated with a thin and short corpus callosum.

Following a comprehensive treatment regimen, including anti-seizure medications, nutritional supplementation, and rehabilitation training, the motor function improved, whereas epilepsy occurred once or twice a year.

Performing WES for the proband and the parents, the Authors identified in the proband a *de novo* variant c.913A>G (p.T305A) in the *NACC1* gene. As revealed by MutationTransfer analysis, the heterogeneous c.913A>G variant in exon 2 of *NACC1* affected the protein features and resulted in splice site changes. The molecular structure differences between the wild type and mutant *NACC1* was demonstrated using PyMOL software. The protein structure of *NACC1* was built and named AFQ96RE7-F1.

The Authors explained the role of *NACC1* in normal neurological function through its involvement in dendritic cells protein turnover and in maintaining synaptic plasticity. They also highlighted the importance of *NACC1* in neural development and its role in various neurodevelopmental and neurodegenerative diseases.

Rozensztrauch et al. studied the quality of life (QOL) of children with trisomy 21 (Down syndrome; OMIM #190685) and the impact of the disorder on family functioning of primary caregivers. Trisomy 21 is the most common genetic disorder causing intellectual disability and is easily diagnosed in resource-limited settings, unlike the diagnostic challenges associated with many other causes of ID (9).

The Authors highlighted the QOL of children with trisomy 21 and the degree of functioning in the domains of physical, emotional, social, school, and psychosocial functioning. The combined scores showed a significant impact of the child’s genetic defect on family functioning, measured by the Family Impact questionnaire, revealing an impaired QOL in children with trisomy 21.

The Authors found a statistically significant relationship between the socioeconomic status of the family and the child’s functioning in the school and emotional functioning domains. The study also highlighted and emphasized that increased QOL of the child with trisomy 21 (Down syndrome) is reflected in an improvement in the QOL of parental and family functioning.

The study of Salih et al. addressed the goals of this Research Topic by identifying the causative novel variant of a rare treatable disease leading to ID. The report by Al-Hassnan et al. also identified a novel ISCA2 missense variant expanding the novel syndrome, multiple mitochondrial dysfunction syndrome 4 (MMDS4, MIM 616370), which they previously identified. On the other hand, Wu et al. reported a novel variant in the NACC1 gene leading to severe ID and demonstrated the molecular structure differences between the wild type and mutant NACC1, paving the way for data sharing and subsequent variant interpretation. The findings of the study by Rozensztrauch et al. ties with the goals of this Research Topic through highlighting a potential prevention program by showing that increased QOL of children with trisomy 21 (Down syndrome) is reflected in improvement in the QOL of their families. However, the studies published in this Research Topic only represent a small sample of the many rare causes of intellectual disability (10).
